# AI-driven optimization of bioremediation strategies for river pollution: a comprehensive review and future directions

**DOI:** 10.3389/fmicb.2025.1504254

**Published:** 2025-04-28

**Authors:** Allen-Adebayo Blessing, Kehinde Olateru

**Affiliations:** ^1^Department of Biological Sciences (Microbiology), College of Natural and Applied Sciences, Okada, Nigeria; ^2^ZeroComplex AI, Lagos, Nigeria

**Keywords:** AI-driven optimisation, bioremediation, river pollution, artificial intelligence, machine learning

## Abstract

This narrative review explores the transformative potential of artificial intelligence (AI) in optimizing bioremediation systems for river pollution control while addressing the challenges and limitations associated with its implementation. The review begins by examining traditional and emerging bioremediation methods, highlighting their limitations and the pressing need for innovative solutions. It then delves into the application of AI technologies in pollution monitoring and bioremediation optimization, providing examples and success stories from existing studies. The challenges of AI-driven bioremediation, including ethical concerns, technological constraints, and the need for responsible deployment, are critically analyzed. Emphasis is placed on fostering interdisciplinary collaboration to overcome these barriers. The review also presents future directions and actionable recommendations, including integrating AI with traditional approaches, addressing technological and policy gaps, and ensuring sustainable management of river ecosystems. Ultimately, this review stresses the revolutionary potential of AI in enhancing bioremediation systems and advocates for urgent action to address the challenges involved, paving the way for sustainable and effective river pollution control strategies.

## Introduction

The growing concern about environmental degradation and its consequences on human health has fueled the quest for creative pollution-reduction strategies (Wani et al., 2024). Artificial intelligence (AI) has emerged as a viable tool to change environmental science (Gupta et al., 2021). This narrative study examines the paradigm change caused by the AI-driven optimization of bioremediation systems for river pollution mitigation, providing a thorough evaluation of current advancements and future perspectives.

AI technologies include a wide range of computational approaches for learning from and making data-driven predictions or choices (Kang et al., 2017). AI has rapidly gained popularity in environmental research owing to its ability to process massive volumes of complicated data, discover patterns, and generate critical insights for tackling environmental issues (Steffi et al., 2022; Russell and Norvig, 2016). From pollution monitoring to remediation, artificial intelligence provides unprecedented opportunities to improve the efficiency and effectiveness of environmental management operations (Steffi et al., 2022). However, it is essential to note that the full potential of AI in bioremediation can only be realized through interdisciplinary collaboration. The complex nature of river pollution requires the expertise of environmental scientists, engineers, policymakers, and other stakeholders to develop comprehensive solutions.

This narrative review focuses centres on the crucial function of bioremediation in river pollution reduction. According to the Environmental Protection Agency (EPA) (2010), Bioremediation, a natural or artificial process that uses microorganisms to break down contaminants, has emerged as a viable and cost-effective method for repairing polluted ecosystems. Rivers, critical freshwater supplies, are vulnerable to various contaminants, including industrial waste, agricultural runoff, and urban sewage, which pose severe challenges to aquatic biodiversity and human well-being (Tao et al., 2021).

Statistics have highlighted the importance of reducing river pollution through innovative approaches. According to the World Health Organization (WHO) (2021), approximately 80% of worldwide wastewater worldwide is discharged into the environment untreated, polluting rivers and worsening waterborne diseases. Furthermore, the United Nations Environment Programme (UNEP) (2017) states that more than 80% of wastewater in underdeveloped countries is dumped into rivers without proper treatment, thereby increasing the risk of waterborne sickness and environmental degradation.

Given the daunting challenges of river pollution, the potential of AI-driven optimization of bioremediation systems is inspiring (Tao et al., 2021; Bansal and Jha, 2023). This technology empowers researchers and practitioners to develop tailored treatments that maximize pollutant removal and minimise ecological disruption. Using AI algorithms to assess environmental data, forecast pollutant behaviour, and optimise bioremediation methods, we can envision a future where river pollution is effectively managed, and our ecosystems thrive.

This narrative review aims to synthesise the existing literature on AI-driven optimisation of bioremediation techniques for river pollution, highlighting significant accomplishments, challenges, and prospects in this emerging topic. We investigated the synergistic integration of AI and bioremediation techniques from an interdisciplinary perspective to provide insights into novel approaches for long-term river ecosystem restoration and human health protection.

### Current bioremediation strategies

Bioremediation, which includes both natural and artificial techniques, is critical for repairing river-polluted ecosystems (Allen-Adebayo et al., 2024). According to Curiel-Alegre et al. (2022), traditional bioremediation approaches such as bioaugmentation and biostimulation have been widely used for decades; however, they have significant limitations. In contrast, modern bioremediation techniques promise to overcome these limitations and provide innovative solutions for long-term pollution management (Das and Chandran, 2011). Traditional bioremediation approaches generally rely on introducing microorganisms to break down contaminants or increase the metabolic activity of existing microbial communities (Curiel-Alegre et al., 2022). Bioaugmentation, for example, involves the introduction of specialized microbial strains capable of metabolizing target pollutants, thereby accelerating pollutant breakdown (Das and Chandran, 2011). Similarly, biostimulation encourages indigenous microorganisms by adding nutrients, oxygen, or electron acceptors to improve their pollutant-degrading capacity (Atlas and Philp, 2005).

However, typical bioremediation technologies have significant drawbacks that restrict their effectiveness in reducing river pollution (Allen-Adebayo et al., 2024). One fundamental difficulty is the limited efficiency of the introduced microbial consortia in complex environmental matrices with varying physicochemical conditions (Head et al., 2006). In addition, the sluggish rate of pollutant degradation and partial transformation of pollutants into innocuous byproducts reduce the efficacy of standard bioremediation procedures, extend remediation time, and perhaps create secondary environmental consequences (Steffi et al., 2022). In contrast, modern bioremediation techniques use cutting-edge technologies and novel approaches to overcome the limitations of existing procedures (Das and Chandran, 2011; Patowary et al., 2023). For example, advanced oxidation processes (AOPs) use chemical reactions involving hydroxyl radicals to destroy various resistant contaminants (Gogate and Pandit, 2004). Nanotechnology-based bioremediation systems use designed nanoparticles to improve pollutant sorption, microbial activity, and degradation kinetics, resulting in higher efficiency and selectivity than traditional methods (Guerra et al., 2018). Recent research has shown that AI-driven optimization can improve new bioremediation systems by allowing for real-time monitoring, adaptive control, and predictive modelling of remediation processes (Steffi et al., 2022). By combining AI algorithms with bioremediation methodologies, researchers can improve pollution removal efficiency, reduce environmental effects, and maximize resource use in a new era of precision environmental management (Gupta et al., 2021). Although classic bioremediation technologies, as shown in Table 1, have long been used to reduce river pollution, their limitations necessitate the investigation of new strategies. The combination of AI-driven optimisation and new bioremediation technologies holds promise for overcoming these problems, paving the way for more effective and sustainable responses to river pollution.

**Table 1:** Various applications of AI in bioremediation research and approaches.

AI application	Bioremediation approach	Description	References
Pollution Monitoring	Data-Driven Monitoring	AI algorithms analyse data from sensors and remote sensing to identify pollution hotspots.	Wani et al. (2024); Tao et al. (2021)
Microbial Selection	Bioaugmentation	Machine learning models predict the best microbial strains for degrading specific pollutants.	Patowary et al. (2023); Gupta et al. (2021)
Optimising Nutrient Supplementation	Biostimulation	Genetic algorithms optimise nutrient addition to enhance microbial activity in contaminated sites.	Sahu et al. (2023); Bibri et al. (2024)
Heavy Metal Removal	Constructed Wetlands	AI frameworks optimise constructed wetlands for efficient heavy metal remediation.	Qasaimeh (2003); Gupta et al. (2021) Biswas et al. (2024)
Predicting Pollutant Transport	Sediment and Water Flow Prediction	Neural networks predict sediment and pollutant transport patterns for targeted interventions.	Tao et al. (2021); Sun and Scanlon (2019)
Real-Time Adaptive Control	Dynamic Bioremediation	Reinforcement learning adjusts treatment parameters dynamically based on real-time data.	Naveed et al. (2024); Chang et al. (2015)
Nanotechnology Integration	Nanoparticle-Enhanced Bioremediation	AI models design and deploy nanomaterials for targeted pollutant degradation.	Patowary et al. (2023); Guerra et al. (2018)
AI-Powered Autonomous Vehicles	Autonomous Monitoring	AI-equipped AUVs map pollutant plumes and collect water samples for precise cleanup strategies.	Anand et al. (2024); Gupta et al. (2021)
Enhanced Wastewater Treatment	AI-Optimized Constructed Wetlands	Machine learning predicts nutrient removal efficiency in constructed wetlands.	Sahu et al. (2023); Qasaimeh (2003)
Policy and Decision Support Systems	Environmental Decision-Making	AI tools support policymakers by predicting the long-term impacts of bioremediation strategies.	Liu et al. (2022); Floridi et al. (2018). Ukpaka (2017)

**Figure 1:**
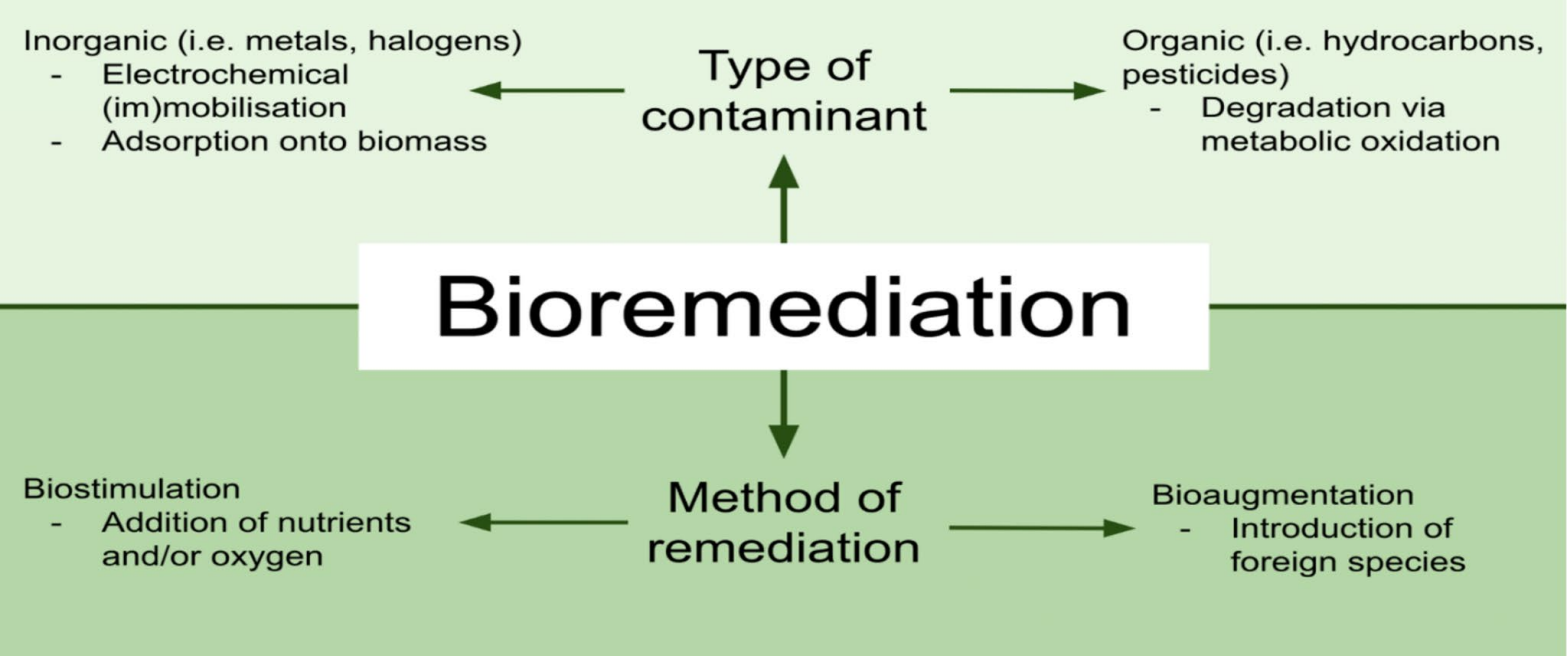
Bioremediation strategies. Source: Western University Tech Review.

Schematic representation of bioremediation strategies based on contaminant type and remediation methods. Figure 1 categorises contaminants as inorganic (e.g., metals, halogens) or organic (e.g., hydrocarbons, pesticides), highlighting specific remediation mechanisms such as electrochemical (im)mobilisation and adsorption onto biomass for inorganic contaminants and metabolic oxidation for organic contaminants. Remediation methods are further divided into biostimulation, involving the addition of nutrients or oxygen to enhance native microbial activity, and bioaugmentation, which involves the introduction of foreign microbial species to improve pollutant degradation efficiency. This framework emphasises the tailored approach required for practical bioremediation of diverse environmental pollutants.

### Application of AI in bioremediation

**Figure 2:**
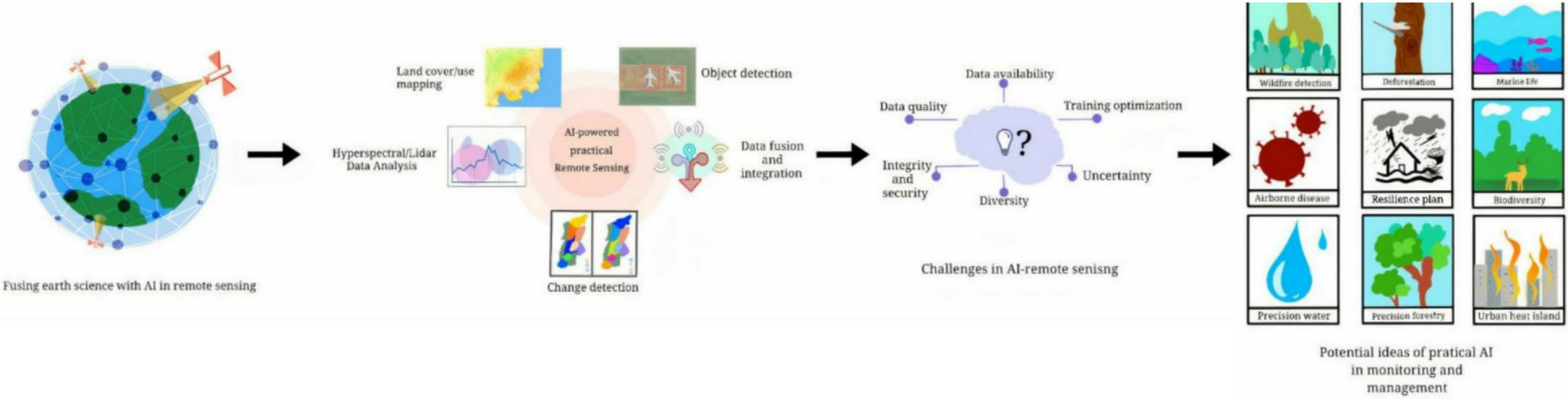
AI in remote sensing. Source: MDPI.

According to Patowary et al. (2023), integrating artificial intelligence (AI) technology has enormous potential for transforming bioremediation systems targeted at reducing river pollution. AI shows excellent potential in data-driven pollution monitoring systems and AI-driven bioremediation process optimization (Liu et al., 2022). By leveraging the capabilities of AI algorithms, researchers can improve the efficiency, accuracy, and sustainability (Bansal and Jha, 2023). Data-driven pollution monitoring systems use artificial intelligence algorithms to analyze vast amounts of environmental data acquired from diverse sources such as remote sensing, sensor networks, and water quality monitoring stations (Liu et al., 2022), as shown in Figure 2. AI methods such as machine learning and neural networks can recognise spatial and temporal patterns in pollutant concentrations, allowing for early detection of pollution hotspots and trends (Sun and Scanlon, 2019). AI-powered pollution monitoring systems integrate multiple datasets to provide complete insights into the dynamics of river pollution, allowing stakeholders to make informed decisions regarding cleanup tactics and resource allocation (Wani et al., 2024).

Furthermore, the AI-driven optimization of bioremediation processes presents an excellent opportunity to improve the efficacy and efficiency of pollutant removal from river ecosystems (Steffi et al., 2022). Researchers can use AI algorithms to optimize critical parameters, such as microbial consortia selection, nutrient supplementation, and process conditions, to maximize pollutant degradation rates while minimizing resource consumption and environmental impacts (Gupta et al., 2021). AI-based optimization methods, such as genetic algorithms and particle swarm optimisation, allow the exploration of complex solution spaces and the identification of optimal remediation strategies based on unique environmental circumstances and pollution profiles (Sahu et al., 2023). In contrast to classic empirical methodologies, AI-driven optimisation techniques provide a data-driven, adaptive approach to bioremediation that considers the dynamic character of environmental systems and the inherent unpredictability of pollutant degradation processes (Liu et al., 2022). AI-powered bioremediation systems can dynamically adapt operational parameters and treatment procedures as environmental circumstances change, thereby ensuring optimal performance (Bansal and Jha, 2023). Overall, the use of AI in bioremediation shows considerable promise in enhancing pollution monitoring and remediation efforts in river ecosystems.

The workflow begins with hyperspectral/LiDAR data analysis to generate outputs such as land cover/use mapping, object detection, and change detection. These outputs are enhanced through data fusion and integration, enabling practical applications of AI in remote sensing. Challenges such as data quality, availability, training optimization, uncertainty, integrity, security, and diversity are identified as critical factors for successful implementation. The right panel illustrates potential applications of practical AI, including wildlife detection, deforestation monitoring, marine ecosystem analysis, airborne disease tracking, resilience planning, biodiversity assessment, precision agriculture, water security, and urban heat island management, showcasing the versatility of AI in addressing complex environmental issues.

### Types of algorithms used in AI for bioremediation

Artificial Intelligence (AI) employs a range of algorithms to optimise bioremediation processes by analysing environmental data, predicting pollutant behaviour, and enhancing remediation strategies (Gupta et al., 2021). These algorithms fall into several categories, each suited to specific applications in environmental management.

1) *Machine Learning Algorithms*: Supervised learning algorithms, such as support vector machines (SVMs) and decision trees, are frequently used to analyse water quality and predict pollutant levels. For unsupervised learning, clustering methods such as k-means help identify pollution hotspots, whereas dimensionality reduction techniques such as Principal Component Analysis (PCA) aid in simplifying complex datasets (Curiel-Alegre et al., 2022). A decision tree describes graphically the decisions to be made, the events that may occur, and the outcomes associated with combinations of decisions and events. It is a tree structured algorithm where nodes represent decision rules and leaves represent outcomes, this is quite easy to interpret, although it is less effective with complex data (Ukpaka, 2017). Their primary advantage lies in their simplicity and interpretability, which makes them accessible to researchers and practitioners. However, they may struggle with capturing highly complex or nonlinear relationships in data, limiting their use in more intricate bioremediation scenarios.
2) *Neural Networks*: Deep learning algorithms, including convolutional neural networks (CNNs) and recurrent neural networks (RNNs), are pivotal for analyzing spatial and temporal pollution data (Wani et al., 2024). Artificial neural networks (ANN) mimic the brain function through interconnected neurons. They have the advantage of being highly adaptable, able to process complex data sets, identify patterns and make predictions. These algorithms excel in processing large datasets from sensors and satellite images, enabling the real-time monitoring of pollutants. A major disadvantage is the requirement for significant computation resources and being prone to overfitting (Biswas et al., 2024)).
3) *Optimization Algorithms*: Genetic algorithms (GAs) and particle swarm optimization (PSO) are widely used to optimise microbial consortia selection, nutrient supplementation, and process parameters for pollutant degradation (Steffi et al., 2022). These algorithms efficiently explore solution spaces to identify the optimal remediation strategies. A significant advantage of Genetic Programming is its ability to generate explicit mathematical models from data, providing users with interpretable results. This is particularly valuable in bioremediation, where understanding pollutant degradation kinetics or environmental interactions is critical. However, this approach can be computationally intensive and may produce models that are complex to interpret without expert knowledge.
4) *Reinforcement Learning*: Adaptive control systems leveraging reinforcement learning dynamically adjust bioremediation parameters based on environmental feedback, ensuring sustained efficiency despite changing conditions (Allen-Adebayo et al., 2024). This approach is well-suited for adaptive control systems in bioremediation, where environmental conditions are dynamic and require real-time adjustments. Its primary advantage is its ability to optimise processes continuously based on feedback. However, reinforcement learning often requires extensive training data and is highly sensitive to the design of the reward functions, which can influence outcomes significantly.

### Interpretative frameworks in AI-driven bioremediation

The interpretative frameworks are important in improving the transparency and predictability of machine learning (ML) approaches in different fields. It assists in understanding the workings of machine learning algorithms and hence enhances confidence in the predictions made. These interpretative frameworks, which include LIME, SHAP, and InterpretML, have been used in making models into very interpretable insights by explaining artificial neural networks (ANN) and support vector machines (SVM), making them easy to use and understand.

1) *SHAP (SHapley Additive exPlanations):* SHAP assigns importances of each input feature to a model’s output using Shapley values which are the measurements from cooperative game theory. This provides a way of giving equal importance to all features while explaining their importance. In Bioremediation, SHAP has been applied to determine the most important ecological parameters that affect the microbial degradation rate. For instance, SHAP was applied to a Random Forest model that was developed to predict the removal of heavy metals in wetlands. The analysis results indicated that nutrients and microbial diversity were the most crucial factors that contributed to effective remediation processes (Lamane et al., 2024). Another study incorporated SHAP with XGBoost to model the estuarine water quality and thus proved the flexibility of the model in various ecological conditions (Lamane et al., 2024).
2) *LIME (Local Interpretable Model-agnostic Explanations):* LIME generates simple, local surrogate models to predict the behaviour of complex ML models. These surrogates provide intuitive explanations for individual predictions. LIME has been used on ANN models to predict pollutant concentration predictions. With the help of LIME, the importance of parameters such as temperature and pH has been established and the experimental designs have been enhanced. For example, Ribeiro et al. applied LIME to show how it can be employed for identifying the most important factors that affect the performance of water quality models.
3) *InterpretML:* InterpretML is a comprehensive framework offering tools for both global and local interpretability, including Explainable Boosting Machines (EBMs) and SHAP. This framework has been utilised to evaluate SVM and Random Forest models in pollutant transport studies. By elucidating the influence of hydrological and chemical parameters, InterpretML has informed effective cleanup strategies in river ecosystems (Nori et al., 2019).

### Case studies and success stories

The use of artificial intelligence (AI) in river pollution remediation has produced encouraging results, with multiple case studies demonstrating the effectiveness of AI-driven solutions for increasing pollutant removal efficiency and recovering aquatic ecosystems (Wani et al., 2024; Allen-Adebayo et al., 2024; Chang et al., 2015). Researchers have shown that using AI algorithms for real-time monitoring, adaptive control, and optimisation of bioremediation processes significantly improves pollutant degradation rates and environmental consequences (Patowary et al., 2023).

A significant example of AI application in river pollution remediation is the use of AI-powered autonomous underwater vehicles (AUVs) to monitor water quality and detect pollutants (Anand et al., 2024). Researchers have created advanced sensor networks using AI algorithms capable of detecting and mapping polluted plumes in real-time (Chang et al., 2015). AI-powered AUVs provide a rapid and precise assessment of pollution levels by autonomously navigating river systems, collecting water samples at crucial places, supporting targeted cleanup operations, and early intervention to minimize environmental impact (Anand et al., 2024).

Additionally, the AI-driven optimization of bioremediation procedures has considerably improved pollutant removal efficiency while decreasing remediation costs. For example, researchers have used machine learning algorithms to optimize the selection and deployment of microbial consortia to degrade specific contaminants in contaminated river sediments (Sun and Scanlon, 2019). AI models can forecast the best mix of microbial species and treatment settings to maximise pollutant degradation rates by assessing environmental data and microbial community dynamics (Naveed et al., 2024).

Several success stories in the literature show how AI-driven solutions improve pollutant removal efficiency. Qasaimeh (2003) used an AI-based optimization framework to bioremediate a heavy metal-contaminated river environment. By combining AI algorithms with bioremediation models, researchers can significantly reduce pollutant concentrations and quickly enhance water quality (Anand et al., 2024). Similarly, Gupta et al. (2021) and Qasaimeh (2003) found that AI-driven optimization improved the efficiency of created wetlands for treating agricultural runoff and reducing nutrient pollution in river systems.

Despite these advances, there are still obstacles to converting AI-driven solutions from research to reality and scaling them up for large-scale river pollution cleanup operations (Tao et al., 2021). Data availability, model robustness, and stakeholder participation are essential considerations for the effective deployment and long-term viability of AI-driven bioremediation initiatives (Bansal and Jha, 2023). In addition, Genome-scale modelling (GSM) and systems biology integrated with machine learning techniques represent advanced approaches for optimizing bioremediation processes. These methodologies allow researchers to model and predict the behaviour of microbial communities under varying environmental conditions, enabling the design of high-performance microbial factories for pollutant degradation.

Systems biology provides a comprehensive framework for understanding the complex interactions within microbial communities. By integrating omics data, such as genomics, transcriptomics, and proteomics, machine learning algorithms can identify key metabolic pathways and regulatory networks that are essential for pollutant breakdown. Genome-scale metabolic models (GEMs) are particularly useful for simulating microbial metabolism and predicting optimal conditions for pollutant degradation. For example, GEMs have been used to enhance the performance of *Pseudomonas putida* in the breakdown of hydrocarbons in contaminated water systems.

Machine learning enhances GSM by identifying critical parameters that influence microbial activity. For instance, supervised learning algorithms can optimise nutrient supplementation, whereas clustering methods aid in grouping microbial strains with complementary metabolic functions. These approaches have succeeded in bioaugmentation strategies for removing persistent organic pollutants.

### Challenges and limitations

Although the incorporation of artificial intelligence (AI) into bioremediation has enormous promise for improving pollution remediation efforts, many obstacles and constraints must be addressed to enable the ethical and effective implementation of AI-driven solutions. Ethical considerations in AI-driven bioremediation include concerns about environmental justice, socioeconomic repercussions, and unexpected consequences (Steffi et al., 2022). As AI technologies become more prevalent in environmental management, concerns arise about fair access to advanced bioremediation methods and the distribution of benefits and dangers across different populations (Chang et al., 2015). Floridi et al. (2018) discussed the ethical difficulties that may arise regarding using AI algorithms to make decisions with potentially far-reaching ecological and societal consequences, raising concerns about accountability, transparency, and democratic governance in environmental decision-making processes.

Technological challenges and data availability impede the mainstream adoption of AI-driven bioremediation techniques (Asquith et al., 2012). Although AI algorithms require vast amounts of high-quality data to train and evaluate predictive models, gathering comprehensive environmental datasets can be difficult, particularly in distant or resource-constrained areas (Liu et al., 2022). Also, Bibri et al. (2024) state that the interoperability of data sources and data format compatibility provide technical challenges to combining varied datasets from multiple sources, limiting the efficiency of AI-powered pollution monitoring and remediation systems. Addressing these difficulties necessitates interdisciplinary collaboration and novel solutions that prioritise ethical considerations, encourage data sharing and openness, and ensure an equal distribution of the advantages and dangers of AI-driven bioremediation interventions (Bansal and Jha, 2023). Researchers can develop socially, economically, and environmentally sustainable AI-driven bioremediation strategies by adopting a participatory approach that includes stakeholders from various backgrounds, such as local communities, policymakers, and industry representatives (Gupta et al., 2021).

Moreover, advances in data analytics, sensor technologies, and remote sensing techniques have provided an opportunity to overcome technological constraints while improving the quantity and quality of environmental data for AI-driven bioremediation applications (Sagan et al., 2020; Yang et al., 2022). Researchers can solve data privacy and security problems by embracing emerging technologies, such as blockchain and federated learning, as well as facilitating data sharing and collaboration among stakeholders (Naveed et al., 2024). Overall, while AI-driven optimization has the potential to revolutionize bioremediation tactics for river pollution, ethical concerns and technological challenges must be carefully addressed to ensure the responsible and effective deployment of AI-powered solutions. By incorporating ethical principles, encouraging interdisciplinary collaboration, and harnessing emerging technologies, researchers can overcome these obstacles and pave the way for a more sustainable and equitable approach to environmental management.

### Future directions and recommendations

The future of AI-powered bioremediation holds significant promise for enhancing river pollution management initiatives through innovation, integration, and legislative changes. Researchers and stakeholders may design a road for more effective, sustainable, and equitable solutions to river pollution by harnessing emerging technologies, encouraging interdisciplinary collaboration, and prioritizing policy initiatives (Tao et al., 2021). Potential developments in AI-driven bioremediation include a variety of technological innovations aimed at increasing pollution removal efficiency, lowering environmental impacts, and improving decision-making processes (Liu et al., 2022; Chang et al., 2015). Future research directions, ranging from developing autonomous robotic systems for in-situ pollutant remediation to applying advanced machine learning algorithms for predicting pollutant fate and transport, can revolutionize environmental management (Gupta et al., 2021). Furthermore, advancements in nanotechnology, biotechnology, and sensor technologies have provided opportunities to improve the specificity, selectivity, and sensitivity of bioremediation techniques, allowing for targeted interventions based on pollutant profiles and environmental conditions (Manjakkal et al., 2021; Ometto et al., 2014).

Strategies for merging AI with traditional bioremediation methods constitute another area for future research and development. Researchers can develop hybrid techniques that combine the strengths of AI algorithms with the practical knowledge and experience of environmental practitioners. For example, implementing AI-powered predictive modelling in decision support systems for pollution source identification and remediation prioritisation can improve the efficiency and effectiveness of traditional cleanup procedures (Steffi et al., 2022).

Policy implications and research goals are critical for determining the future of river pollution control and environmental management (Ometto et al., 2014). Policymakers are advised to prioritize investments in research, infrastructure, and capacity-building efforts to promote the responsible use of AI-powered bioremediation technology (Floridi et al., 2018). Furthermore, legal frameworks and governance mechanisms must be modified to meet the high speed of technological innovation while maintaining environmental integrity, human health, and social equality (Patowary et al., 2023).

Interdisciplinary studies that combine ecological, social, and technological aspects to establish holistic pollution management systems are among the research goals for improving river pollution control (Wani et al., 2024). Furthermore, initiatives to improve data sharing, standardisation, and interoperability are critical for optimizing the utility of AI-powered bioremediation systems and fostering stakeholder engagement (Steffi et al., 2022). Finally, future directions and recommendations for the AI-driven optimization of bioremediation solutions for river pollution include technological advances, integration tactics, and legislative initiatives that promote innovation, collaboration, and sustainability (Tao et al., 2021). By implementing these recommendations, researchers and policymakers can realize the revolutionary potential of AI-driven bioremediation to address the complex challenges of river pollution and protect freshwater ecosystems for future generations.

## Conclusion

In conclusion, this narrative review thoroughly investigates the revolutionary potential of AI-driven optimisation in bioremediation systems to reduce river pollution. An assessment of the present bioremediation approaches, use of AI technology, case studies, obstacles, and prospects reveals that AI has enormous potential for changing pollution control efforts in river ecosystems. From data-driven pollution monitoring to AI-driven remediation process optimization, integrating AI algorithms provides opportunities to improve pollutant removal efficiency, reduce environmental impacts, and promote sustainable river management practices. However, ethical concerns, technological barriers, and regulatory ramifications must be addressed to ensure the effective deployment of AI-powered solutions. Interdisciplinary collaboration, new research, and policy initiatives will be critical in influencing the future of AI-powered bioremediation and protecting freshwater ecosystems for future generations.
